# Conservative Treatment for Bilateral Displaced Proximal Humerus Head Fracture

**DOI:** 10.7759/cureus.657

**Published:** 2016-06-27

**Authors:** Ruy E Rodriguez-Corlay, Ricardo Velutini-Becker, Luis D Aguilar-Alcalá

**Affiliations:** 1 Orthopedics, ABC Medical Center

**Keywords:** proximal humeral head, fracture, displaced, conservative treatment, bilateral

## Abstract

Proximal humerus fracture represents five to eight percent of all fractures and is twice as common in women than in men. Most cases of displaced fracture of the proximal humerus are treated surgically; it is probable that more cases are preferred to be treated surgically greater than required. The optimal treatment for these fractures remains controversial, but physicians have a tendency to treat via open reduction and fixation with angular locking plates or glenohumeral arthroplasty.

We present a case of a 71-year-old woman with bilateral displaced proximal humeral fracture. Conservative treatment was initiated with two hanging casts, achieving radiological reduction on week one. After two additional weeks of casting, treatment continued with radiologic control and home physical therapy, ultimately an excellent functional outcome and adequate radiological reduction was obtained.

Even in bilaterally displaced proximal humerus fractures, conservative treatment can be an efficient option, reducing complications, reaching adequate functional results and acceptable radiographic reduction.​

## Introduction

Proximal humerus fracture represents five to eight percent of all fractures and is twice as common in women than in men. The majority of these fractures are nondisplaced or nonangulated, and 89.1% can be managed in a conservative way [1].

However, displaced proximal humerus fractures are mostly treated surgically [2]; three or four displaced fragments, as well as the fractures associated with dislocation, are considered complex, and fortunately only represent five percent of the total proximal humeral fractures [3].

The proximal humerus is a frequent location for fragility fractures. In patients older than 65, a proximal humerus fracture is the third most commonly indicated fracture behind hip and distal radius fractures, always found in association with other morbidities that produce up to three months of physical disability [4].

This kind of fracture has a bimodal incidence; in young patients, they are the result of high-energy trauma, and in the elderly they are associated with a moderate energy event after falling due to low bone quality. The optimal treatment on 3- and 4-fragment fractures remains controversial, with a clear tendency for physicians to perform open reduction and internal fixation with angular locking plates or glenohumeral arthroplasty [5].

This case report emphasizes that a conservative treatment in displaced proximal humeral fractures is still a viable and good option for any patient, even in a bilateral presentation.

The authors have obtained the patient's informed written consent for print and electronic publication of this case report.

## Case presentation

A 71-year-old female patient was presented to the emergency department after falling from her own height with a direct contusion on both hands, with the wrist and elbows in extension. She had a past medical history which was positive for arterial hypertension (treated with Captopril) and rheumatoid arthritis (treated with Leflunomide).

**Figure 1:**
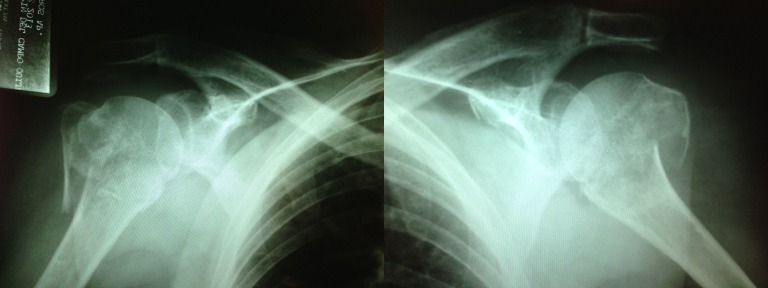
Simple x-ray AP bilateral views

Her fall resulted in localized pain in both shoulders, and limited range of motion leading to an inability to perform activities with her arms raised above the head. X-rays were taken on an anteroposterior (AP) bilateral view (Figure 1), and she was diagnosed with a Neer III bilateral displaced proximal humerus fracture.

Simple X-rays are the most commonly used diagnostic method for a proximal humerus fracture, and often two different views are necessary to analyze the pattern of a fracture. AP and lateral views should be taken. In cases of complex fractures or uncertainty based on the x-rays, a computed tomography (CT) scan can be used.

**Figure 2:**
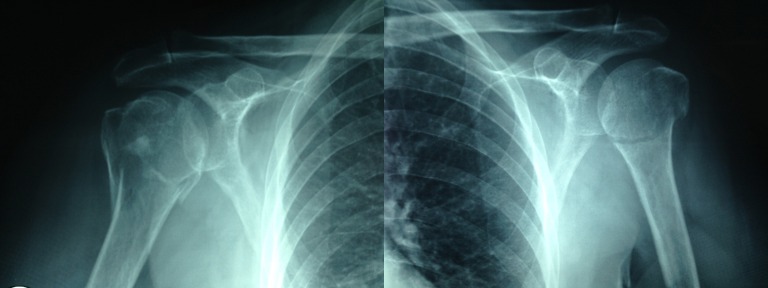
Simple x-ray AP bilateral views, week 1

After a clinical evaluation of the patient, though there was no contraindication for surgical treatment, conservative treatment was chosen to address the pathology. Two hanging casts were placed using two slings on both arms. A week later, a control x-ray was taken showing a reduction of some of the fragments (Figure 2).

**Figure 3:**
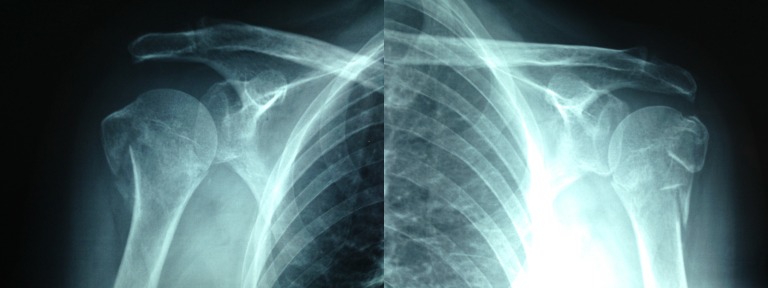
Simple x-rays AP bilateral views, week 3

At week three of treatment, a new x-ray was taken showing even more bone fragment reduction when compared to week one (Figure 3). Clinically, the patient showed consolidation with partial radiographic consolidation, so the casts were removed. She continued to use the bilateral slings and initiated pendulum movements for the following three weeks at home. She initiated active movements at week six.

**Figure 4:**
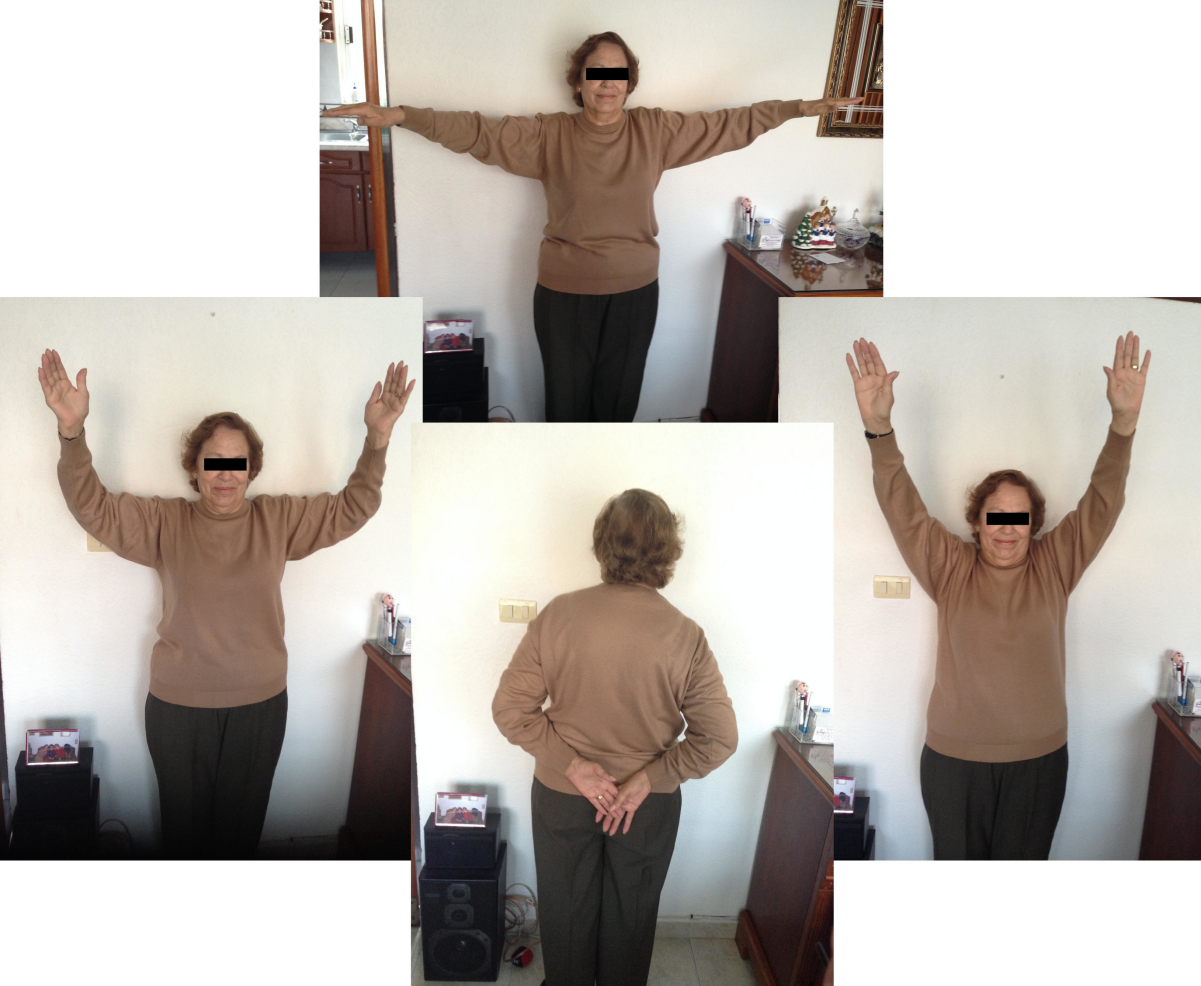
Clinical range of movements, week 7

At the week seven follow-up consultation, she arrived with only mild pain during any activity behind the back during active internal rotation on both arms. The bilateral ranges of motion were as follows: flexion, 100°; extension, 15°; internal rotation, 100°; external rotation, 90°; abduction, 100°; adduction, 30°; muscle strength, 5/5; with no alteration of the distal neurovascular status (Figure 4). We obtained the Disabilities of the Arm, Shoulder and Hand (DASH) Score of 16/100 (100 representing the worst-case scenario).

## Discussion

The proximal humerus is a frequent location for fragility fractures, and in patients older than 65, a proximal humerus fracture is the third most common fracture behind hip and distal radius fractures. Most proximal humerus fractures with minimal displacement can be treated in a conservative way.

According to literature, displaced 2-, 3- or 4-bone fragment fractures need surgical treatment to optimize the anatomical consolidation of the fracture, with the main objective of regaining the natural humeral retroversion and the relation between the tuberosities and the head. This is due to a demonstrated increase in the deformity of the fracture during the consolidation time in elderly patients and those with a greater initial displacement of the fracture [6].

However, standard surgical treatment i.e., open reduction and internal fixation using stable angular plates and cerclages, has not shown functional differences after 12- and 24-month follow-ups. Such differences have not been observed even on displaced 3-part proximal humerus fractures treated with conservative treatment or open reduction internal fixation with angular stability plates at 24 months [7,8]. In one study, standard surgical treatment showed no significant difference in health-related quality of life EuroQol-5D (EQ-5D), Constant Score, and Disabilities of the Arm, Shoulder and Hand (DASH) at 24 months [9]. Another study reported surgical treatment of four-part displaced proximal humerus fractures with hemiarthroplasty yielded improved EQ-5D compared with the conservative treatment, but offered no significant difference in the functional scores [10].

Conservative treatment is considered appropriate for any fracture in patients older than 75 with limited functional expectations, due to the expected quality of pain relief combined with little functional limitation. If the functional expectations are higher, or dislocation is present, surgical treatment should be considered.

We presented a case in which the displaced fracture occurred in both shoulders simultaneously, and we were able to treat the patient in a conservative way for both humeral fractures. It is important to highlight that it is not a highly tolerable treatment because the patient is in acute pain and has no mobility in either arm. Therefore, to complete the treatment, the patient must be very cooperative and have an integral home support system due to the functional disability during treatment

## Conclusions

The majority of 3- or 4-part displaced fractures in elderly patients with low functional requirements can be treated with conservative treatment, yielding adequate pain relief and a limited but good functional outcome. More complex four-part fractures associated with dislocation, unbearable pain or high functional expectations (i.e., as seen in younger patients) may have better outcomes with surgical management. With proper adaptations, despite the trends in the literature, even in displaced bilateral three part proximal humerus fractures, the conservative treatment can be used as definitive treatment with an adequate functional outcome.
